# Inhibition of *Escherichia coli* O157:H7 and *Salmonella enterica* Isolates on Spinach Leaf Surfaces Using Eugenol-Loaded Surfactant Micelles

**DOI:** 10.3390/foods8110575

**Published:** 2019-11-15

**Authors:** Songsirin Ruengvisesh, Chris R. Kerth, T. Matthew Taylor

**Affiliations:** 1 Department of Nutrition and Food Science, Texas A&M University, College Station, TX 77843-2253, USA; songsirin@gmail.com; 2 Department of Animal Science, Texas A&M University, College Station, TX 77843-2471, USA; c-kerth@tamu.edu

**Keywords:** micelles, plant-derived antimicrobial, Enteric pathogens, leafy greens

## Abstract

Spinach and other leafy green vegetables have been linked to foodborne disease outbreaks of *Escherichia coli* O157:H7 and *Salmonella* *enterica* around the globe. In this study, the antimicrobial activities of surfactant micelles formed from the anionic surfactant sodium dodecyl sulfate (SDS), SDS micelle-loaded eugenol (1.0% eugenol), 1.0% free eugenol, 200 ppm free chlorine, and sterile water were tested against the human pathogens *E. coli* O157:H7 and *Salmonella* Saintpaul, and naturally occurring microorganisms, on spinach leaf surfaces during storage at 5 °C over 10 days. Spinach samples were immersed in antimicrobial treatment solution for 2.0 min at 25 °C, after which treatment solutions were drained off and samples were either subjected to analysis or prepared for refrigerated storage. Whereas empty SDS micelles produced moderate reductions in counts of both pathogens (2.1–3.2 log_10_ CFU/cm^2^), free and micelle-entrapped eugenol treatments reduced pathogens by >5.0 log_10_ CFU/cm^2^ to below the limit of detection (<0.5 log_10_ CFU/cm^2^). Micelle-loaded eugenol produced the greatest numerical reductions in naturally contaminating aerobic bacteria, *Enterobacteriaceae*, and fungi, though these reductions did not differ statistically from reductions achieved by un-encapsulated eugenol and 200 ppm chlorine. Micelles-loaded eugenol could be used as a novel antimicrobial technology to decontaminate fresh spinach from microbial pathogens.

## 1. Introduction

The U.S. Centers for Disease Control and Prevention (CDC) has estimated that 47.8 million cases of foodborne illnesses occur annually in the U.S. due to known and unspecified foodborne disease agents [1]. Of these pathogens, *Escherichia coli* O157:H7 and non-typhoidal *Salmonella enterica* serotypes were deemed responsible for approximately 63,153 cases [2] and 1,027,561 cases of domestically acquired foodborne illnesses, respectively [3]. From 2006 to 2017 in the U.S., the numbers of foodborne disease cases associated with the shiga toxin-producing *E. coli* (STEC), and the various serovars of the non-typhoidal salmonellae, associated with fresh fruits and vegetables, has increased [4,5]. This increase could be partly due to improved surveillance for human pathogens [6], increased consumption of raw or minimally processed produce items, as well as other contributing factors (e.g., use of nontreated biological soil amendments or pathogen-contaminated irrigation water, and other practices which could increase pathogen transmission risks). Among many commodities, spinach and other leafy greens have been associated with multiple *E. coli* O157:H7 human disease outbreaks [7,8,9]. While less frequently associated with leafy greens in the U.S., multiple outbreaks of leafy green disease outbreaks involving multiple *Salmonella* spp. have been reported across many industrialized nations, summarized recently by Chaves et al. [10]. Foodborne disease outbreaks can cause substantial economic losses including medical expenses, lost wages, damage control costs for product recall and disposal of affected products, and production time loss [11].

Essential oils and their components (EOCs) are volatile, hydrophobic substances that can be extracted from various parts (e.g., flowers, leaves, rhizome, seeds, fruits, wood, and bark) of aromatic plants) [12]. Essential oils contain bioactive components that are derivatives of alcohols, ketones, aldehydes, esters, and phenols [12]. It has been reported that EOCs possess insecticidal, antioxidant, anti-inflammatory, anti-allergenic, anticancer, and antimicrobial properties, thereby potentially beneficial in medical, pharmaceutical, and food industries [13]. In foodstuffs, however, high concentrations of EOCs are often required to inactivate microorganisms due to the hydrophobic nature of some EOCs [14,15]. For example, eugenol is water-soluble up to only 4.93 g/L, though it is miscible in alcohols such as ethyl alcohol [16]. The requirement for use of elevated concentrations of EOCs can render EOCs impractical as food additives or sanitizers, as they may be excessively costly at usage concentrations and/or impart undesirable flavor and/or aroma to the food product [17,18]. Encapsulation has, therefore, been recommended for improving upon these negative characteristics of plant-derived antimicrobial agents, by increasing water-dispersibility, reduce the required dosage needed for foodborne pathogen inhibition, and provide protection to the antimicrobial agent from rapid volatilization [19,20,21]. Weiss et al. [14], in their review of nanoencapsulation strategies for food antimicrobials delivery to foods, recommended that encapsulating materials be inexpensively procured to offset the cost of additional processing needed to form the encapsulated structure. In this case, sodium dodecyl sulfate (SDS) can be purchased relatively inexpensively, and manufacture of micelles does not require highly costly equipment. In addition, consumer use of produce rinsing in the home prior to consumption would reduce the potential for undesirable flavor or mouthfeel consequences on micelle-treated produce surfaces. Thus, delivery methods for EOCs can be utilized to improve antimicrobial activities of EOCs in food systems so as to reduce the content of EOC required for antimicrobial functionality without significant compromise to sensory acceptability of treated commodities.

To enhance delivery of EOCs to microorganisms in foodstuffs, surfactants can be utilized to encapsulate EOCs [18,22,23]. Surfactants are surface-active, amphiphilic molecules that contain both hydrophilic and hydrophobic components; they can be classified as anionic, cationic, zwitterionic, or nonionic [24]. At low concentrations, surfactants adsorb to the aqueous phase of a lipid/water interface, lowering the surface tension [25]. When present at or above the critical micelle concentration (CMC), surfactant molecules will aggregate to form thermodynamically favored structures known as micelles. In micelle structures, hydrophobic molecules such as EOCs can be encapsulated inside the hydrophobic core, while hydrophilic headgroups of surfactants face outwardly contacting the aqueous phase [24,26].

In several studies, efficient pathogen inactivation using EOCs-encapsulated surfactant micelles/emulsion in foodstuffs has been reported [18,22,23,27]. Nonetheless, limited studies have been conducted to evaluate the antimicrobial activities of EOCs-containing micelles on the surfaces of fresh produce for the purpose of pathogen decontamination. Thus, the main objective of this study was to determine the efficacy of eugenol-loaded surfactant micelles, compared to other antimicrobial treatments, specifically non-encapsulated eugenol and 200 ppm free chlorine, to reduce numbers of inoculated *E. coli* O157:H7 and *S.* Saintpaul on surfaces of spinach leaves stored refrigerated. The second objective was to evaluate the efficacy of eugenol-containing micelles to reduce numbers of microbial hygiene indicator on leaf surfaces during refrigerated storage.

## 2. Materials and Methods

### 2.1. Preparation of Antimicrobial Micelles and Other Treatments

Eugenol-loaded micelles and other treatments (free eugenol, empty micelles, 200 ppm free chlorine, sterile distilled water) were prepared in identical manner to methods reported previously by our group [28]. Briefly, eugenol stock solution (70% *w/v*) was prepared by dissolution of eugenol (Sigma-Aldrich Co., St. Louis, MO, USA) in 95% ethyl alcohol (Koptec, King of Prussia, PA, USA), and stored at 5 °C until ready for use. Sodium dodecyl sulfate (SDS) micelles (1.0% *w/v*) were produced containing eugenol at 1.0% EOC according to previous methods [29]. After stirring until optical density at 632 nm stabilized, micelles were filter-sterilized by filtering through a 0.45 µm cellulose acetate filter. Micelles were then stored at 5 °C for no more than 36 h prior to use.

### 2.2. Revival of Bacterial Pathogens and Preliminary Assessment of Consistent Overnight Pathogen Growth for Pathogen Cocktail Preparation

Rifampicin-resistant (Rif^R^; 100.0 µg/mL) *E. coli* O157:H7 (Strain K3999) from the pathogen isolate recovered from a 2006 U.S. spinach-borne disease outbreak and *S. enterica* serovar Saintpaul (Strain FDA/CFSAN 476398) from the 2008 U.S. peppers-transmitted disease outbreak were selected for spinach sample inoculation and decontamination. Pathogens were revived from cryo-storage (−80 °C) in the culture collection of the Food Microbiology Laboratory (Department of Animal Science, Texas A&M University, College Station, TX, USA) individually inoculating each isolate into a sterile 10.0 mL volume of Tryptic Soy Broth (TSB; Becton, Dickinson and Co., Franklin Lakes, NJ, USA) and incubating for 24 h at 35 °C without shaking. After incubation, a sterile loop was used to collect 10.0 µL of each culture; each was then aseptically passed into a new sterile 10.0 mL volume of TSB. These were subsequently incubated for 24 h at 35 °C. Following the second passage of cultures to complete revival and activation, equal volumes of microorganisms were blended into a cocktail for spinach surface inoculation, targeting an inoculation of approximately 6.0 log_10_ CFU/cm^2^. Preliminary tests were completed prior to experimental startup to verify researchers’ ability to consistently produce predictable numbers of pathogen isolates following 24 h incubation in TSB at 35 °C, in order to reliably produce an inoculum. Following incubation of microorganisms, TSB volumes of each pathogen were serially diluted in 0.1% (*w/v*) peptone (Thermo-Fisher Scientific, Waltham, MA, USA) diluent and enumerated on Tryptic Soy Agar (TSA; Becton, Dickinson and Co.). Following 24 h incubation of inoculated TSA Petri plates at 35 °C, plates were counted and counts were log_10_-transformed. The experiment was replicated in identical manner three times (*n* = 3) and numbers of each organism compared to one another to confirm that one pathogen would not contribute significantly more cells to the cocktail than the other. A cocktail of Rif^R^ *E. coli* O157:H7 and *S.* Saintpaul was subsequently prepared for spinach inoculation according to the method of Cálix-Lara et al. [30] without modification.

### 2.3. Antimicrobial Activity Testing for Antimicrobial Treatments on Pathogens-Inoculated and Noninoculated Spinach Leaf Samples Held under Refrigeration

Unwashed, freshly harvested spinach was purchased from a local fruit and vegetable distributor and transported immediately in insulated coolers containing cooling pouches to the Food Microbiology Laboratory (Department of Animal Science, Texas A&M University, College Station, TX, USA). For each sample, three pieces, each 10 cm^2^, of spinach were aseptically excised using sterile scalpel and borer, placed in an empty sterile Petri dish, and spot-inoculated with approximately 7.0 log_10_ CFU/mL cocktailed Rif^R^ *E. coli* O157:H7 and *S*. Saintpaul. Pathogen cocktail was spotted onto samples (ten spots at 10.0 μL), after which pathogen-inoculated samples were air-dried at ambient temperature (25 ± 1 °C) for 1.0 h to allow pathogen attachment to spinach leaf surfaces.

To test the sanitizing/growth inhibition efficacy of each treatment on pathogens or naturally occurring hygiene microorganisms, encapsulated eugenol (1.0% SDS + 1.0% eugenol-loaded micelles), free eugenol (1.0% eugenol), empty micelles (1.0% SDS), 200 ppm chlorine (adjusted to pH 7.0 with 0.1 N HCl), and sterile distilled water were individually applied to inoculated spinach samples in Petri dishes by immersing in 20 mL of treatment solution. Positive controls (pathogen inoculated without any treatment or non-inoculated spinach samples used for testing antimicrobial/sanitizing treatments against background microbiota) and negative controls (uninoculated sample without treatment) were included to determine pathogen attachment to spinach surfaces and confirm no naturally occurring 100.0 µg/mL Rif^R^ microbes, respectively. For day 0 samples, encapsulated eugenol, free eugenol, empty micelles, chlorine, and sterile distilled water were individually applied to Petri dishes via 2 min immersion with 20 mL of treatment solution, after which the solution was drained off and spinach samples immediately transferred to a sterile stomacher bag and mixed with 99 mL 0.1% (*w/v*) peptone diluent by pummeling in a stomacher (230 rpm) for 1 min.

For all non-day 0 samples, treatments were applied to spinach leaf samples in identical manner as for day 0-assigned samples, drained of treatment solution, and then transferred to new sterile Petri dishes, where they were stored at 5 ± 1 °C covered in saran film to afford oxygen transmission under dark conditions. Samples were withdrawn after 3, 5, 7, or 10 days of refrigerated storage for subsequent enumeration of inoculated pathogens or naturally occurring microbial organisms. As with day 0 samples, to enumerate pathogens, samples were placed in stomacher bags and pummeled with 99 mL of 0.1% peptone diluent for 1 min. Pummeled samples were serially diluted in 9 mL of 0.1% peptone diluent and dilutions were spread on surfaces of Lactose-Sulfite-Phenol Red-Rifampicin (LSPR) agar supplemented with 100.0 µg/mL rifampicin, in order to differentially enumerate *E. coli* O157:H7 colonies (cream-white with halo of fermented lactose) from *S.* Saintpaul colonies (black-centered colonies with no halo of lactose fermentation) [31]. Following 24 h incubation at 35 °C, colonies of Rif^R^ *E. coli* O157:H7 and *S.* Saintpaul were counted and recorded.

For enumeration of naturally occurring microbiota (aerobic bacteria, *Enterobacteriaceae*, and yeasts and molds) from non-inoculated, antimicrobial-treated spinach surface samples, resulting samples were serially diluted in 99 mL sterile 0.1% peptone diluent and 1.0 mL volumes were spread on 3M^TM^ Petrifilm^TM^ Aerobic Count Plates, 3M^TM^ Petrifilm^TM^ Enterobacteriaceae Count Plates, and 3M^TM^ Petrifilm^TM^ Yeast and Mold Count Plates. Aerobic Count Plate and Enterobacteriaceae Count Plate petrifilms were each incubated at 35 °C for 48 h, while Yeast and Mold Count Plate petrifilms were incubated at 25 °C for 5 days, all according to manufacturer instructions. Colonies were counted after incubation.

### 2.4. Statistical Analysis of Data

For preliminary data gathered for pathogen cocktail preparation (Section 2.2), mean counts of each pathogen (*n* = 3) were compared to one another by unpaired *t*-test (2-tailed, *p* = 0.05). All spinach decontamination experiments (Section 2.3) were replicated thrice identically; two independent samples were completed for each sample/treatment combination within a replicate (*n* = 6). The experiment was designed and completed as a full factorial, with α = 0.05; spinach samples were randomly assigned to antimicrobial treatment and storage period conditions at experiment outset. All microbiological plate count data were log_10_-transformed prior to statistical analysis. The limit of detection for plating assays was 0.5 log_10_ CFU/cm^2^. In cases where microbial numbers were below the limit of detection, the value of 0.4 log_10_ CFU/cm^2^ was inserted for purposes of comparison of mean microbial counts by treatment and storage period. Log_10_-transformed counts of each pathogen, or microbial hygiene indicator group, were compared for the main effects of antimicrobial treatment, storage period, and their interaction by a two-way analysis of variance (ANOVA). Statistically differing mean microorganism counts (pathogens, hygiene indicator grouping) were separated by Tukey’s Honestly Significant Differences test at *p* = 0.05. Statistical analysis was completed on JMP Pro v.14 for Macintosh (SAS Institute, Inc., Cary, NC, USA).

## 3. Results

### 3.1. Consistency of Overnight Growth of Salmonella Saintpaul and E. coli O157:H7 Organisms for Cocktail Preparation

Mean populations of *E.coli* O157:H7 and *Salmonella* Saintpaul isolates following 24 h incubation at 35 °C during preliminary trials (Section 2.2) were 7.4 ± 0.2 and 7.6 ± 0.1 log_10_ CFU/mL, respectively. Mean plate counts of the pathogens following growth were not different from one another by *t*-test (*p* = 0.156), and were thus assessed to not provide non-differing counts of each pathogen to cocktail preparations for subsequent experiments on spinach leaves.

### 3.2. Inhibition of Salmonella Saintpaul on Spinach Surfaces by Antimicrobial Treatments over 10 Days of Refrigerated Storage

Table 1 presents the least-squares means of *Salmonella* Saintpaul populations on spinach leaf surfaces following treatment with SDS micelle-encapsulated eugenol, free eugenol, empty SDS micelles, 200 ppm chlorine, or sterile distilled water. For *Salmonella* reduction on spinach surfaces, overall, the trend of antimicrobial effects from greatest to least was Encap = Free-Eug ≥ 200 HOCl > SDS-Mic ≥ DW. Encapsulated eugenol, free eugenol, and chlorine exerted efficient residual effects in reducing pathogen populations to below or just over detectable levels after day 0 of storage. Only the free and micelle-encapsulated eugenol treatments reduced pathogens to below the limit of detection by plating (0.5 log_10_ CFU/cm^2^). The population on the positive control (inoculated, nontreated) on day 0 of storage was 6.0 log_10_ CFU/cm^2^. On day 0, populations of *S*. Saintpaul after treatment with encapsulated eugenol, free eugenol, empty micelles, chlorine, and sterile water were varied, ranging from 1.8 to 5.6 log_10_ CFU/cm^2^. Early in the experiment, free eugenol was equally effective as chlorine at reducing the pathogen on spinach, and produced a greater numerical reduction than did encapsulated eugenol in reducing *S*. Saintpaul (though counts of surviving pathogen between treatments did not differ). Conversely, neither empty SDS micelles nor sterile water reduced populations of *S*. Saintpaul (*p* ≥ 0.05) on day 0 (Table 1). From days 3 until 10, all treatments resulted in *S*. Saintpaul declining in a treatment and time-specific manner, ultimately ranging at day 10 of storage from 0.4 to 4.7 log_10_ CFU/cm^2^ (Table 1). Micelle-encapsulated eugenol, free eugenol, and 200 ppm chlorine were similarly effective in reducing *S.* Saintpaul populations and were more effective than empty SDS micelles and sterile water at days 3 through 10. Encapsulated eugenol and free eugenol initially reduced the pathogen compared to the control, and inhibited pathogen growth to undetectable numbers continuously from days 3 to 10. Compared to the control, water treatment increased the population of *S.* Saintpaul to 4.7 log_10_ CFU/cm^2^ on day 10. Compared to the level of *S.* Saintpaul on day 0, the levels of *S.* Saintpaul on the positive control decreased from day 5 to 10 of storage (*p* < 0.05), likely the result of cold temperature storage in combination with potential for pathogen cells to be exposed to spinach-derived compounds with antimicrobial activity (e.g., organic acids, phytoaxelins, phenolic compounds).

### 3.3. Inhibition of E. coli O157:H7 on Spinach Surfaces by Antimicrobial Treatments over 10 Days of Refrigerated Storage

Similar trends were observed for *E. coli* O157:H7-inoculated spinach treated with antimicrobials (free, encapsulated) as those reported for *Salmonella*-inoculated spinach (Section 3.2). Table 2 depicts populations of *E. coli* O157:H7 on spinach samples after antimicrobial sanitizing treatment, over 10 days of refrigerated (5 ± 1°C) storage. The initial population of *E. coli* O157:H7 on the positive control on day 0 was 6.0 log_10_ CFU/cm^2^. On day 0, antimicrobial treatments, except sterile water, reduced populations of *E. coli* O157:H7 to numbers ranging from 2.3 to 5.0 log_10_ CFU/cm^2^. As was the case with *Salmonella* Saintpaul testing, initially free eugenol treatment produced the greatest numerical reduction in pathogen counts. Moreover, similar to *Salmonella* testing, encapsulated eugenol-treated *E. coli* O157:H7 counts did not differ from those of the free eugenol-treated *E. coli* O157:H7 count, though numerical counts of *E. coli* O157:H7 were higher than like counts of *Salmonella* at day 0 for free and micelle-loaded eugenol treatments. From days 3 to 10, *E. coli* O157:H7 populations treated with either micelle-encapsulated or free eugenol bore non-detectable pathogen counts (0.4 log_10_ CFU/cm^2^). Conversely, other treatments (sterile water, empty SDS micelles, and 2 00 ppm chlorine) produced smaller reductions in pathogen counts following their application. Encapsulated eugenol, free eugenol, and chlorine reduced pathogen counts to non-detection or near non-detection values within 7 days of refrigerated storage (*p* ≥ 0.05); all were more effective than empty micelles or water (*p* < 0.05) on day 3. From days 5 to 10, all treatments but sterile water reduced populations of *E. coli* O157:H7 to lower levels than positive controls (*p* < 0.05). The levels of *E. coli* O157:H7 on untreated spinach samples decreased from 6.0 to 4.0 log_10_ CFU/cm^2^ from day 0 to 10, a similar but less substantial decline as that observed for *S.* Saintpaul (Table 1 and Table 2).

### 3.4. Inhibition of Naturally Occurring Microbial Hygiene Indicator Groups on Treated Spinach over 10 Days of Refrigerated Storage

**Figure 1:**
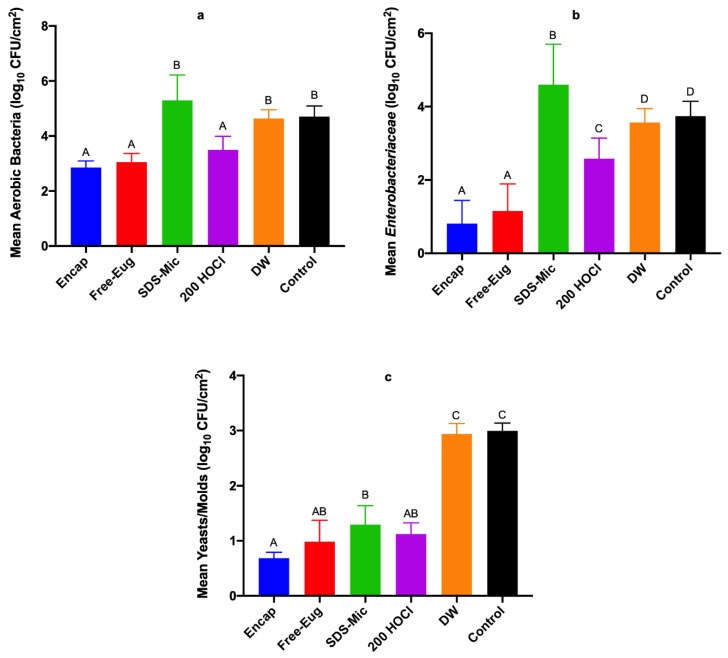
Means of naturally occurring microorganisms on spinach samples as function of antimicrobial treatment: (**a**) aerobic bacteria, (**b**) *Enterobacteriaceae*, and (**c**) yeasts and molds (*p* < 0.0001). Treatments were: 1.0% sodium dodecyl sulfate (SDS) micelles loaded with 1.0% eugenol (Encap); 1.0% unencapsulated eugenol (Free-Eug); 1.0% SDS micelles unloaded (SDS-Mic); 200 ppm pH 7.0 free chlorine (200 HOCl); sterile distilled water (DW); no treatment, non-inoculated (Control). Bars depict arithmetic means from three identical replications with duplicate independent samples per replicate (*n* = 6); error bars depict one sample standard deviation from the mean. Columns not sharing capitalized letters (A, B, C, D) differ at *p* = 0.05.

With respect to antimicrobial treatments and their impacts on naturally contaminating hygiene-indicating microorganisms, for aerobic bacteria and *Enterobacteriaceae*, treatments followed the trend from greatest to least antibacterial effects of Encap = Free-Eug ≥ 200 HOCl > DW > SDS-Mic (Figure 1). The antifungal effect of treatments on surfaces of spinach samples followed the trend of Encap = Free-Eug = 200 HOCl ≥ SDS-Mic > DW (Figure 1). In the case of spinach leaf samples that were utilized for determining the efficacy of antimicrobial treatments against naturally contaminating aerobic bacteria, *Enterobacteriaceae*, and fungi (yeasts/molds), microbial loads on spinach samples were significantly influenced by antimicrobial treatment for all groups of tested microorganisms. In all cases, encapsulated and free eugenol reduced organisms versus sterile water and the control, but surviving counts of aerobic bacteria, *Enterobacteriaceae* and fungi did not differ for micelle-loaded eugenol versus free eugenol (Figure 1). SDS micelles exerted some antimicrobial effect when compared with water or the control for all groups of microbes, though not to the extent observed for eugenol-including treatments or the 200 ppm free chlorine treatment. Indeed, for *Enterobacteriaceae*, SDS micelles appeared to produce a higher count of *Enterobacteriaceae* versus the control and water-treated samples, potentially resulting from de-clumping of cells by the surfactant, or higher initial loads on SDS micelles-treated spinach samples at the experiment initiation (Figure 1b). While no group of microorganisms was reduced to non-detectable levels, eugenol treatments resulted in the fewest numbers of hygiene indicator microbes on treated spinach, indicating potential for best outcomes related to protection of spinach keeping quality.

## 4. Discussion

Eugenol (4-allyl-2-methoxyphenol) is a naturally occurring phenolic EOC in clove oils and has been reported to exhibit effective antimicrobial activities against a wide range of microorganisms [32,33,34]. Reported mechanisms of action of EOCs against microorganisms have included cellular membrane disruption, alteration in membrane permeability, release of proteins and nucleic acids, and structural and morphological changes [32]. In this study, SDS was utilized to encapsulate 1% eugenol for inhibiting enteric bacterial pathogens and naturally occurring microorganisms on surfaces of spinach samples. SDS, an anionic surfactant, is a derivative of lauric acid and a mixture of sodium alkyl sulfates consisting of a 12-carbon tail attached to a sulfate head group, rendering it amphiphilic [35,36]. The possible functions of surfactant micelles in delivering an antimicrobial to pathogens may include: 1) enhanced dispersion of EOC in aqueous phase; 2) transport of EOCs to microbial membranes, and; 3) disruption of microbial membranes to enhance uptake of EOC [19,37,38,39]. Micelles themselves are covered by polar headgroups, making them amphiphilic structures [40]. However, the surfactant monomers of the micelles structures are amphiphilic and may thermodynamically bind to bacterial membrane components [40]. In this research, the antimicrobial activities of free and encapsulated eugenol did not significantly differ. Although eugenol is hydrophobic, it possesses slight water solubility (0.64 g/L) [41] and thus may have resulted in partial dissolution and dispersion of eugenol in wash water.

The rough surfaces of spinach [42], as well as cracks, pockets, crevices, and native openings (e.g., stomata), may favor microbial attachment and provide protection to microorganisms from antimicrobial intervention [43,44]. On leaf surfaces, there is a boundary layer, a thin layer of air influenced by the leaf surface [45]. The layer can vary in thickness and can influence the temperature, moisture, and speed of water vapor leaving the stomata through the motionless layer [45]. When spinach samples were treated with encapsulated or free eugenol, the antimicrobial EOC may have become trapped in a boundary layer and crevices. During storage, eugenol may have vaporized and exerted residual effect in inactivating microorganisms. The surface of spinach is covered with cuticle, a continuous extracellular membrane of polymerized lipids with associated waxes [46]. The hydrophobic nature of the waxy cuticle may have prevented chlorine, which is more hydrophilic, from inactivating microorganisms on spinach surfaces.

Hypochlorous acid (HOCl) is the principal form of available chlorine in an aqueous solution that exerts the greatest bactericidal activity against a wide range of microorganisms. To maintain available HOCl, the pH of the solution must be maintained in the range of 6.0 to 7.5 [47]. In this study, the pH of a chlorine solution was adjusted to 7.0 at the experiment’s outset, prior to its application onto inoculated samples. Distilled water was used to prepare the chlorine solution, so the presence of organic matter was reduced. Thus, chlorine showed potent antibacterial effect in reducing pathogens and microbiota on fresh produce in the study. Indeed, chlorine treatment was as effective as eugenol-including treatments in the cases of aerobic bacteria and yeasts/molds but not for *Enterobacteriaceae*, wherein counts of microbes treated with 200 ppm chlorine did not statistically differ versus those treated either with micelle-loaded or free eugenol. Effects of chlorine on microbial inactivation in leafy greens have been reported throughout many refereed papers and expert reports. Zhang and Farber [48] reported the maximum log_10_ reduction of *L. monocytogenes* at 4 and 22° C to be 1.3 and 1.7 log_10_ CFU/g for lettuce and 0.9 and 1.2 log_10_ CFU/g for cabbage, respectively. In the current study, chlorine (200 ppm) produced greater reductions for inoculated pathogens versus naturally occurring *Enterobacteriaceae* (Table 1 and Table 2; Figure 1), similar to results reported by other researchers testing 100–200 ppm HOCl on spinach [49,50], potentially resulting from differences in differing attachment strengths from naturally occurring versus inoculated pathogen cells, as well as potential for naturally occurring cells to locate effectively into protected niches on the leaf surface [51]. Erkman [52] reported that 10 ppm HOCl (pH 7.0) applied via immersion with agitation for 5 min reduced *E. coli* on lettuce, parsley, and pepper by 1.2, 1.6, and 2.6 log_10_ CFU/mL, respectively. Nevertheless, in produce packing operations, accumulation of organic matter (e.g., field soil, debris, fruit, leaves) in a dump tank or flume water, as well as alkaline pH of wash water, can decrease effectiveness of chlorine [47,53].

In this study, micelle-loaded eugenol produced the highest numerical reductions in naturally contaminating aerobic bacteria, *Enterobacteriaceae*, and fungi, although with the exception of the *Enterobacteriaceae*, these did not differ statistically from reductions achieved by un-encapsulated eugenol and 200 ppm chlorine. It was reported that *Enterobacteriaceae* and pseudomonads are predominant on surfaces of leafy greens [45]. Thus, increased populations of aerobic bacteria and *Enterobacteriaceae* on spinach surfaces in this study could have been due to the ability of these bacteria to metabolize or tolerate SDS [54,55,56]. Kramer et al. [55] reported that 200 strains of independent isolates of *Enterobacteriaceae* members (e.g., *E. coli*, *Shigella flexneri*, *Shigella sonnei*, *Salmonella* Arizonae, *Klebsiella pneumoniae*, etc.) were highly tolerant to SDS and were able to grow in the presence of ≥5% SDS. In contrast, previous research has indicated that SDS demonstrated antimicrobial activity against foodborne fungal microbes, inhibiting colony development and mycotoxin synthesis [57,58].

Utilization of EOC-encapsulating micelles or emulsions for inactivation of pathogens on fresh produce surfaces has been reported. Park et al. [59] reported clove bud oil (0.02%) + benzothoium chloride (0.002%) emulsion inactivated inoculated *S.* Typhimurium and *Listeria monocytogenes* on fresh-cut pak choi by 1.9 to 2.0 log_10_ CFU/g, respectively. Kang et al. [22] showed that cinnamon leaf essential oil in cetylpyridinium chloride produced 1.8 and 1.5 log_10_ CFU/g reductions against *L. monocytogenes* and *E. coli* O157:H7, respectively; quality of kale leaves was not affected during storage. In our previous study, eugenol (1% *w/v*) encapsulated in SDS (1% *w/v*) micelles were used for inhibition of *S.* Saintpaul and *E. coli* O157:H7 as well as native microbiota on tomato skin surfaces during refrigerated and abuse storage [28]. In that study, antimicrobial effects of free and encapsulated eugenol did not differ from those of HOCl and empty SDS micelles during refrigerated storage. However, reductions in pathogen counts to non-detectable levels were only observed with free and encapsulated eugenol [28]. EOC-encapsulated micelles could be used as an alternative to the commonly used sanitizers to reduce pathogens on fresh produce, potentially achieving greater pathogen reductions versus those typically observed by washing in chlorinated water [60].

## 5. Conclusions

Overall, micelle-encapsulated and eugenol displayed similar efficacies for reducing the enteric bacterial human pathogens *E. coli* O157:H7 and *Salmonella*, as well as for microbial hygiene-indicating microorganisms, on surfaces of spinach leaf samples during a simulated washing and subsequent refrigerated storage. Antimicrobial-loaded micelles may be used as an alternative to conventional antimicrobial technologies for decontaminating surfaces of leafy green produce commodities from microbial pathogens as a means to produce human food safety for consumers of these agricultural commodities.

## Figures and Tables

**Table 1:** Least-squares means of surviving *Salmonella* Saintpaul (log_10_ CFU/cm^2^) on spinach surfaces as a function of the interaction of antimicrobial treatment and days of aerobic storage at 5 °C.

Storage Period (Days)	Encap ^1^	Free-Eug	SDS-Mic	200 HOCl	DW	Control
0	2.8GH ^2^	1.8HI	5.4ABCD	2.0HI	5.6ABC	6.0A
3	0.4K	0.4K	4.7CDEF	0.7JK	5.2ABCD	5.8AB
5	0.4K	0.4K	4.5DEF	1.6IJ	4.8BCDEF	4.5CDEF
7	0.4K	0.5JK	4.0EF	0.9IJK	4.8BCDE	4.3DEF
10	0.4K	0.4K	3.6FG	0.5JK	4.7BCDEF	3.8EFG
*p* ≤ 0.0001	Pooled Standard Error = 0.2					

^1^ Antimicrobial treatments were: 1.0% sodium dodecyl sulfate (SDS) micelles loaded with 1.0% eugenol (Encap); 1.0% un-encapsulated eugenol (Free-Eug); 1.0% SDS micelles unloaded (SDS-Mic); 200 ppm pH 7.0 free chlorine (200 HOCl); sterile distilled water (DW); inoculated, nontreated (Control). ^2^ Values depict least-squares means calculated from three identically completed replicates, each containing duplicate identically processed independent samples (*n* = 6). Means read across columns and rows that do not share capitalized letters (A, B, C, …) differ by two-way analysis of variance and Tukey’s Honestly Significant Differences Means Separation Test at *p* = 0.05.

**Table 2:** Surviving *Escherichia coli* O157:H7 (log_10_ CFU/cm^2^) on spinach surfaces as a function of the interaction of antimicrobial treatment and days of aerobic storage at 5 °C.

Storage Period (Days)	Encap ^1^	Free-Eug	SDS-Mic	200 HOCl	DW	Control
0	3.1DEFG ^2^	2.3GHI	5.0ABC	2.7FGH	5.3AB	6.0A
3	0.4K	0.4K	4.1CDE	0.7JK	4.7ABC	5.9A
5	0.4K	0.4K	3.8CDEF	1.5IJK	4.2BCD	4.6BC
7	0.4K	0.6JK	2.9EFGH	0.8JI	4.1CDE	4.4BC
10	0.4K	0.4K	1.7HIJ	0.6JK	3.9CDEF	4.0CDE
*p >* 0.0001	Pooled Standard Error = 0.3					

^1^ Antimicrobial treatments were: 1.0% sodium dodecyl sulfate (SDS) micelles loaded with 1.0% eugenol (Encap); 1.0% unencapsulated eugenol (Free-Eug); 1.0% SDS micelles unloaded (SDS-Mic); 200 ppm pH 7.0 free chlorine (200 HOCl); sterile distilled water (DW); inoculated, nontreated (Control). ^2^ Values depict least-squares means calculated from three identically completed replicates, each containing duplicate identically processed independent samples (*n* = 6). Means read across columns and rows that do not share capitalized letters (A, B, C, …) differ by two-way analysis of variance and Tukey’s Honestly Significant Differences Means Separation Test at *p* = 0.05.
